# BARMR1-mediated sorafenib resistance is derived through stem-like property acquisition by activating integrin-FAK signaling pathways

**DOI:** 10.1038/s41392-020-0189-8

**Published:** 2020-06-12

**Authors:** Xing Rong Guo, Meng Ye Shan, Yu Huang, Zong Li Zhang, Yue Yuan, Long Jun Dai, Jue Wang, Xue Peng Zhou, Fu Yun Ji, Jun Ming Tang, Zhong Ji Meng, Xu Zhi Ruan

**Affiliations:** 1 Hubei Key Laboratory of Embryonic Stem Cell Research, Taihe Hospital, Hubei University of Medicine, 442000 Shiyan, Hubei China; 2 College of Basic Medicine, Hubei University of Medicine, 442000 Shiyan, Hubei China; 3 Jiangsu Hengrui Medicine Co. Ltd, Lianyungang, China; 4 Department of Neurosurgery, Taihe Hospital, Hubei University of Medicine, Shiyan, China; 5 Department of Surgery, University of British Columbia, Vancouver, BC Canada; 6 Hepatobiliary and Pancreatic Surgery Diagnosis and Treatment Center, Taihe Hospital, Hubei University of Medicine, Shiyan, China; 7 Institute of Biomedical Research, Taihe Hospital, Hubei University of Medicine, Shiyan, China; 8 Department of Infectious Diseases, Taihe Hospital, Hubei University of Medicine, Shiyan, China

**Keywords:** Oncogenes, Cancer stem cells

Dear Editor,

BARMR1 (alternatively named FAM92A1 or FAM92A) gene was first identified in 2002 and is a highly conserved gene and widely expressed in most tissues in mammals.^1^ We first cloned the complete CDS sequence of BARMR1 in 2007,^2^ which encodes a protein with a Bin-Amphiphysin-Rvs (BAR) domain. BAR-containing proteins are known to bind onto lipid membrane surface and generate membrane curvature and have been demonstrated to play diverse roles in cell growth, inflammation and cell migration.^3^

Our previous studies found that BARMR1 was highly expressed in a variety of proliferative cells, such as embryonic stem cells and cancer cells,^2^ and demonstrated that BARMR1 retains many oncogenic properties as evidenced by facilitating cell proliferation and promoting colony formation and cell migration in various in vitro and in vivo cancer studies, including renal cancer^4^ and glioblastoma multiforme cells.^5^ Wang et al. also found that BARMR1 affects cell proliferation in human osteosarcoma cells.^3^ High expression of BARMR1 was negatively correlated with the prognosis of patients with acute myeloid leukemia. Through comprehensive analysis public databases (http://www.cbioportal.org/ and http://gepia2.cancer-pku.cn/), we found that the high expression of BARMR1 was associated with the progression and metastasis in multiple cancers (Supplementary Fig. S1). The Cancer Genome Atlas (TCGA) database shows the high expression of BARMR1 in multiple cancers (Supplementary Fig. S1a, b), which is significantly associated with poor disease-free survival and shorter overall survival in a pan-cancer analysis of 10,891 patients (*p* < 0.0001) (Supplementary Fig. S1c–h). These data encouraged us to broadly and deeply study its role in the development and progression of cancer. We knocked down BARMR1 expression by siRNA in 10 cancer cell lines and 2 non-cancer cell lines, which significantly inhibited cancer cell proliferation, especially in hepatocellular carcinoma cells (Supplementary Fig. S2a). Therefore, we hypothesized that BARMR1 might be a crucial molecule involved in the HCC development.

To test our hypothesis, we analyzed BARMR1 expression in HCC patients’ tissue samples and various human HCC cell lines. The immunohistochemistry and western blot results indicate that BARMR1 expression was significantly up-regulated in HCC tissues compared with normal tissues (Supplementary Fig. S2b, c), and increased in all HCC cell lines with a high degree of malignancy compared with low proliferating and metastatic HCC cell lines and the LO2 normal liver cell line (Supplementary Fig. S2d, e). RTCA data revealed that the upregulated BARMR1 significantly enhanced cell viability (*P* < 0.01) in both HepG2 and MHCC97H cell lines compared to those in the controls. In contrast, BARMR1 knockdown reduced cell proliferation (*P* < 0.01) (Supplementary Fig. S3a, b). However, BARMR1 did not significantly affect cell proliferation of the normal human liver cell line LO2 (Supplementary Fig. S3c). In accordance with in vitro data, the mean tumor size (~2-fold) and weight (~3-fold) of the neoplasms formed with HepG2-BARMR1-OE cells were significantly bigger (*P* < 0.05, Fig. S4a–c) than those of the control, while the tumor size (~0.6-fold) and weight (~0.5-fold) were remarkably reduced in the BARMR1-KD HepG2-derived xenografts compared with those of the controls (Supplementary Fig. S4d–f). In accordance with the tumor size and weight, more proliferating cells were detected in HepG2-BARMR1-OE-derived xenografts determined with Ki-67 assay (*P* < 0.05; Fig. S4g). Apart from promoting HCC cell growth, BARMR1 also regulated metastatic ability by promoting cell migration in vitro (Supplementary Fig. S5a, b) and liver metastasis in vivo (Supplementary Fig. S5c–f). BARMR1 positively regulated EMT in HCC cells through upregulation of N-cadherin, while simultaneously down-regulating E-cadherin expression (Supplementary Fig. S5g). Our current data reported here strongly indicate that BARMR1 exerts oncogenic effects on tumor progression via promoting cell proliferation and by increasing the metastatic abilities in HCC.

To explore the mechanisms underlying BARMR1-regulated tumor progression, BARMR1 protein interacting partners were previously identified through a yeast two-hybrid approach. The interaction between BARMR1 and Galectin-1 (Gal-1) proteins was verified by co-immunoprecipitation (Supplementary Fig. S6a). The expression and activities of related cellular signaling molecules were determined. Our results suggest that the interaction of BARMR1 with Galectin-1 enhanced HCC cell proliferation and the metastasis of HCC cells by activating the H-RAS/ERK and αv/β3 Integrin/FAK pathways (Supplementary Fig. S6b–e).

Studies have shown that Gal-1 regulates sorafenib resistance in HCC. Our studies showed that BARMR1 was involved in HCC cells’ sensitivity to sorafenib in vitro (Fig. 1a, b and Supplementary Fig. S7a). As shown in Fig. 1c–k, sorafenib-induced tumor growth inhibition was exhibited from the 7th day (D21) after the initial application of sorafenib in the orthotopic transplantation nude mice model. Compared with the control mice, the significant promotion of tumor growth were observed on the post-injection day 28th in the group with HepG2 BARMR1-OE cells (*P* < 0.05) (Fig. 1d–g). Meanwhile, the tumor volume and weight of mice treated with sorafenib in the group of BARMR1-KD cells were significantly smaller than those of controls (*P* < 0.05) (Fig. 1h–k), suggesting that BARMR1 led to HCC sorafenib resistance in vitro and in vivo.

**Fig. 1:**
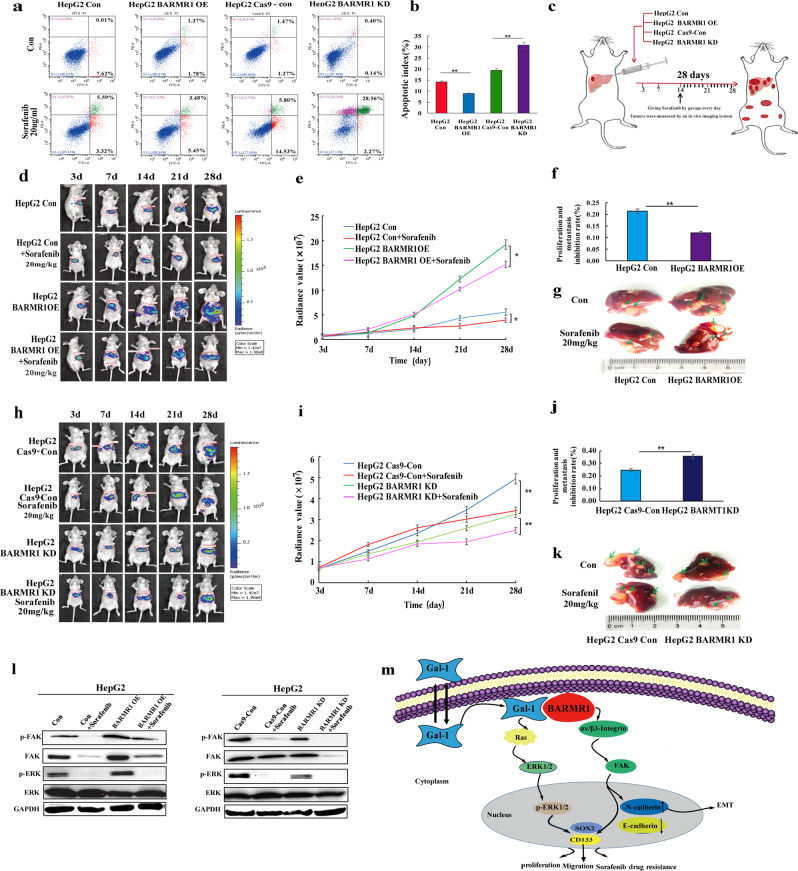
BARMR1-mediated sorafenib resistance is through stem-like property acquisition by activating integrin-FAK signaling pathways. **a** The apoptosis of HepG2 cells treaded with sorafenib was analyzed by flow cytometry. The cell apoptosis was determined with Annexin V fluorescein isothiocyanate (FITC)/PI staining. The percentages of Annexin-V-positive cells were indicated. **b**The apoptotic index was defined as the percentage of apoptotic cells (**P < 0.001 vs. control). **c** Schematic model illustrating the administration time and route of HepG2-Luc cells with overexpression/knockdown of BARMR1 and sorafenib in a mouse orthotopic model. **d–g** The effects of sorafenib in orthotopic hepatocellular carcinomal models with overexpression of BARMR1 were evaluated. **d** Serial pictures taken at different time points. **e** The changes of fluorescein radiance values of the neoplasms formed from different groups. **f** Representative proliferation inhibition rate of orthotopic tumors by sorafenib. **g** Representative images of orthotopic tumors and relevant metastatic tumors (*p < 0.05 and **p < 0.001 vs. control). **h–k** The effects of sorafenib in orthotopic hepatocellular carcinomal models with knockdown of BARMR1 were evaluated. **h** Serial pictures taken at different time points. **i** The changes of fluorescein radiance values of the neoplasms formed from different groups. **j** Representative proliferation inhibition rate of orthotopic tumors by sorafenib. **k** Representative images of orthotopic tumors and relevant metastatic tumors (*p < 0.05 and **p < 0.001 vs. control). **l** After sorafenib treatment (24 h), the protein levels of ERK, p-ERK, FAK, pFAK in HepG2 cells with overexpression or knockdown of BARMR1 were detected by western blotting. **m** Schematic representation of the proposed mechanism of BARMR1 in HCC cells. BARMR1 contributes to tumorigenesis in HCC cells by interacting with Galectin-1, which could activate the H-Ras/ERK and αvβ3-integrin/FAK pathway to up regulate CD133 and OCT4 stem-like property gene expression, thereby promoting proliferation, migrating capacity, and affecting its drug sensitivity of sorafenib in the treatment of HCC

Interestingly, in the HepG2 cells with ectopic expression BARMR1, more tumor-sphere cells were discovered (Supplementary Fig. S7b) and stemness factors CD133 and SOX2 were upregulated (Supplementary Fig. S7c) when compared with those in the controls. We hypothesized that BARMR1 may mediate sorafenib resistance through stem-like property acquisition. Further results showed that BARMR1-mediated sorafenib resistance may be derived through stem-like property acquisition by activating phosphorylated FAK rather than by activating phosphorylated ERK (Fig. 1l).

In summary, BARMR1 is a novel oncogene involved in HCC through increasing cell proliferation, stemness and metastatic abilities. We demonstrated, for the first time, that BARMR1 contributes to tumorigenesis in HCC by interacting with Gal-1. BARMR1-Gal-1 interaction activates ERK and FAK pathways and enhances stem-like property gene expression, thereby affecting sorafenib sensitivity in the treatment of HCC (Fig. 1m). Our data provide functional evidence for BARMR1 to be a therapeutic target for developing an effective treatment for cancer.

## Supplementary information

Supplementary_Materials

SupplementalFigure s1

SupplementalFigure s2

SupplementalFigure s3

SupplementalFigure s4

SupplementalFigure s5

SupplementalFigure s6

SupplementalFigure s7
