# Supplementary material for: BARMR1-mediated sorafenib resistance is derived through stem-like property acquisition by activating integrin-FAK signaling pathways
Source: Signal Transduct Target Ther. 2020 Jun 12;5:97. doi: 10.1038/s41392-020-0189-8 (PMC7293271; doi:10.1038/s41392-020-0189-8)
Supplement: Supplementary file 1 — Supplementary_Materials [file 41392_2020_189_MOESM1_ESM.docx]

**Supplementary Materials for**

**BARMR1-mediated sorafenib resistance is derived through stem-like property acquisition by activating integrin-FAK signaling pathways**

Xing Rong Guo, Meng Ye Shan, Yu Huang, Zong Li Zhang, Yue Yuan, Long Jun Dai, Jue Wang, Xue Peng Zhou, Fu Yun Ji, Jun Ming Tang, Zhong Ji Meng, Xu Zhi Ruan

Contact: Xing Rong Guo, email: gxrdl@126.com; Xu Zhi Ruan email: ruanxuzhi@163.com

**Supplementary information, Figures**

Fig.S1 BARMR1 is frequently amplified and associated with shorter patient survival in cancer.

Fig.S2 BARMR1 is overexpressed in HCC cell lines and human tissues.

Fig.S3. BARMR1 is involved in HCC cell proliferation *in vitro*

Fig.S4. BARMR1 is involved cell proliferation in HepG2-Luc cells *in vivo.*

Fig.S5. BARMR1 is involved in HCC cell migration *in vitro* and *in vivo*.

Fig.S6. BARMR1 activates H-RAS/ERK and αvβ3-integrin/FAK cellular signaling pathways depending on its interaction with Galectin-1

Fig.S7. BARMR1-mediated sorafenib resistance is through stem-like property acquisition.

**Supplementary information, Materials and Methods**


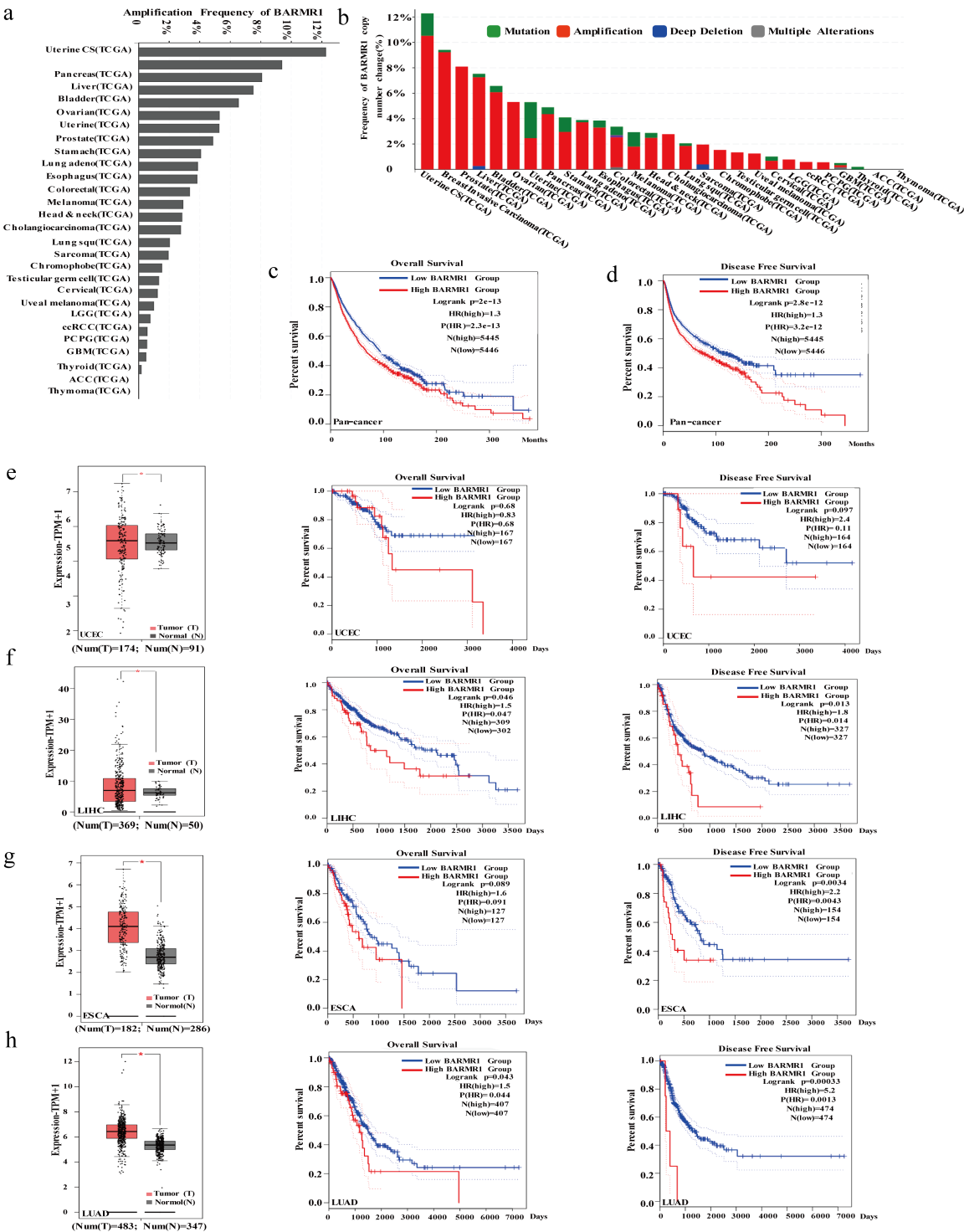


**Fig.S1 BARMR1 is frequently amplified and associated with shorter patient survival in cancer.**

(a) Amplification frequency of BARMR1 across cancers in publically available cancer sequencing database (http://www.cbioportal.org/; http://gepia2.cancer-pku.cn/) of clinical samples.

(b) Copy-number alterations of BARMR1 across cancers in TCGA datasets.

(c&d) Disease free survival and Kaplan–Meier analysis of overall survival curves of BARMR1 clinical outcomes in pan-cancer from the TCGA database.

(e-h) Disease free survival and Kaplan–Meier analysis of BARMR1 expression and clinical outcomes in uterine carcinosarcoma ((UCEC),e) hepatocellular carcinoma ((LIHC),f) esophageal squamous cell carcinoma ((ESCA),g) and lung adenocarcinoma((LUAD),h) from the TCGA database.


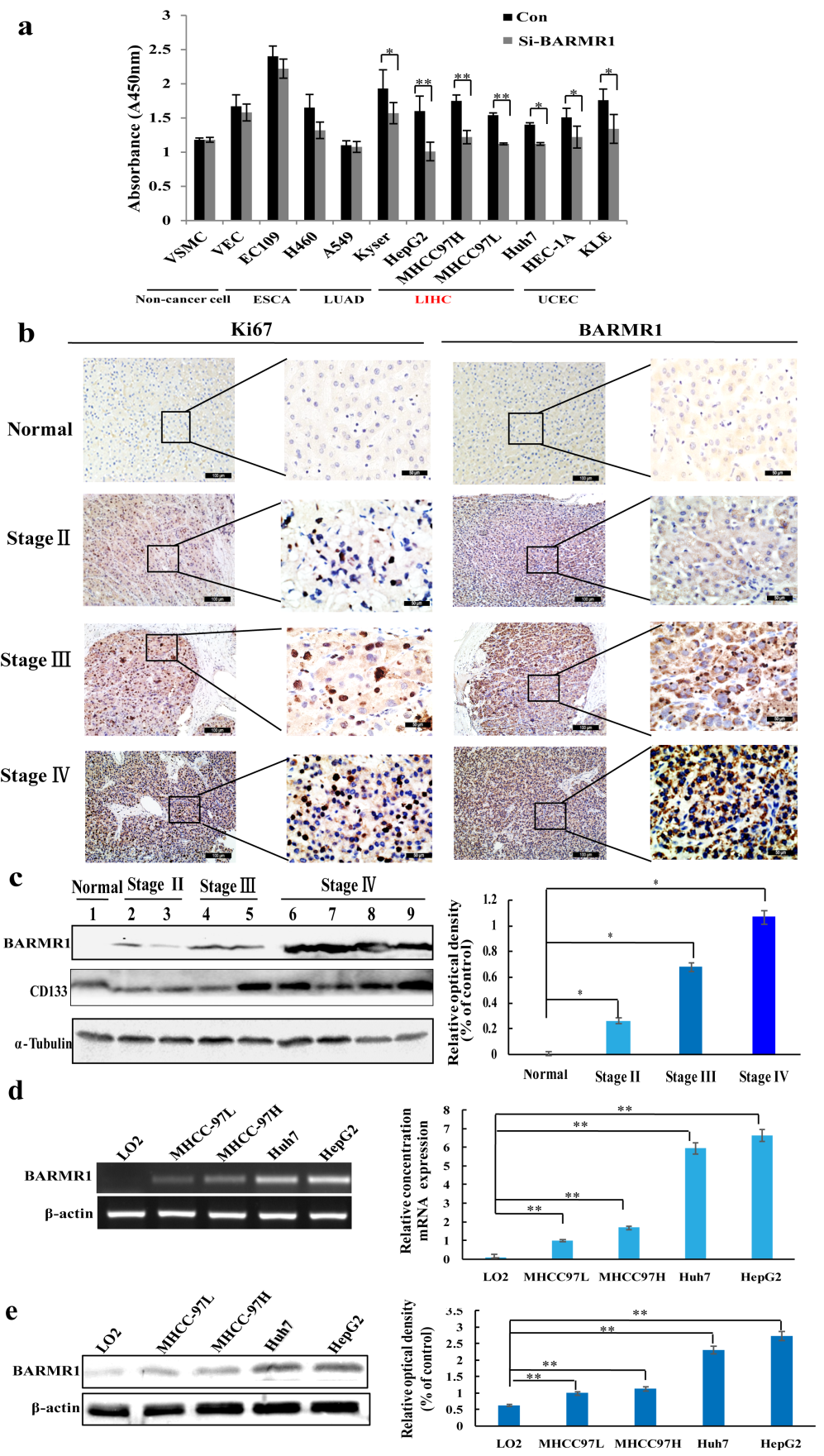


**Fig.S2. BARMR1 is overexpressed in HCC cell lines and human tissues.**

(a) The viability of 10 tumor cell lines and 2 non-cancer cell lines that transfected with siControl or siBARMR1 was analyzed using a CCK-8 assay. The cell growth inhibition at 48 hrs is shown in the bar graph.

(b) High BARMR1 expression in HCC tissues correlated with poor prognosis. Representative immunohistochemistry images of BARMR1 and Ki67 protein expression in HCC tumor tissues and normal brain tissue.

(c) BARMR1 and CD133 proteins in fresh-frozen specimens from normal human hepatocyte and HCC tissues were detected by western blotting.

(d) The mRNA levels of BARMR1 in different malignant degrees of HCC cell lines (MHCC97L,MHCC97H, Huh7, HepG2) and normal hepatocyte cell LO2 were analyzed by qPCR.

(e) The protein levels of BARMR1 in different malignant degrees of HCC cell lines (MHCC97L,MHCC97H,Huh7,HepG2) and normal hepatocyte cell LO2 was determined by western blotting. α-tubulin was used as a loading control. Data expressed± SEM of three independent experiments; *p<0.05 and **p<0.001 vs. control.


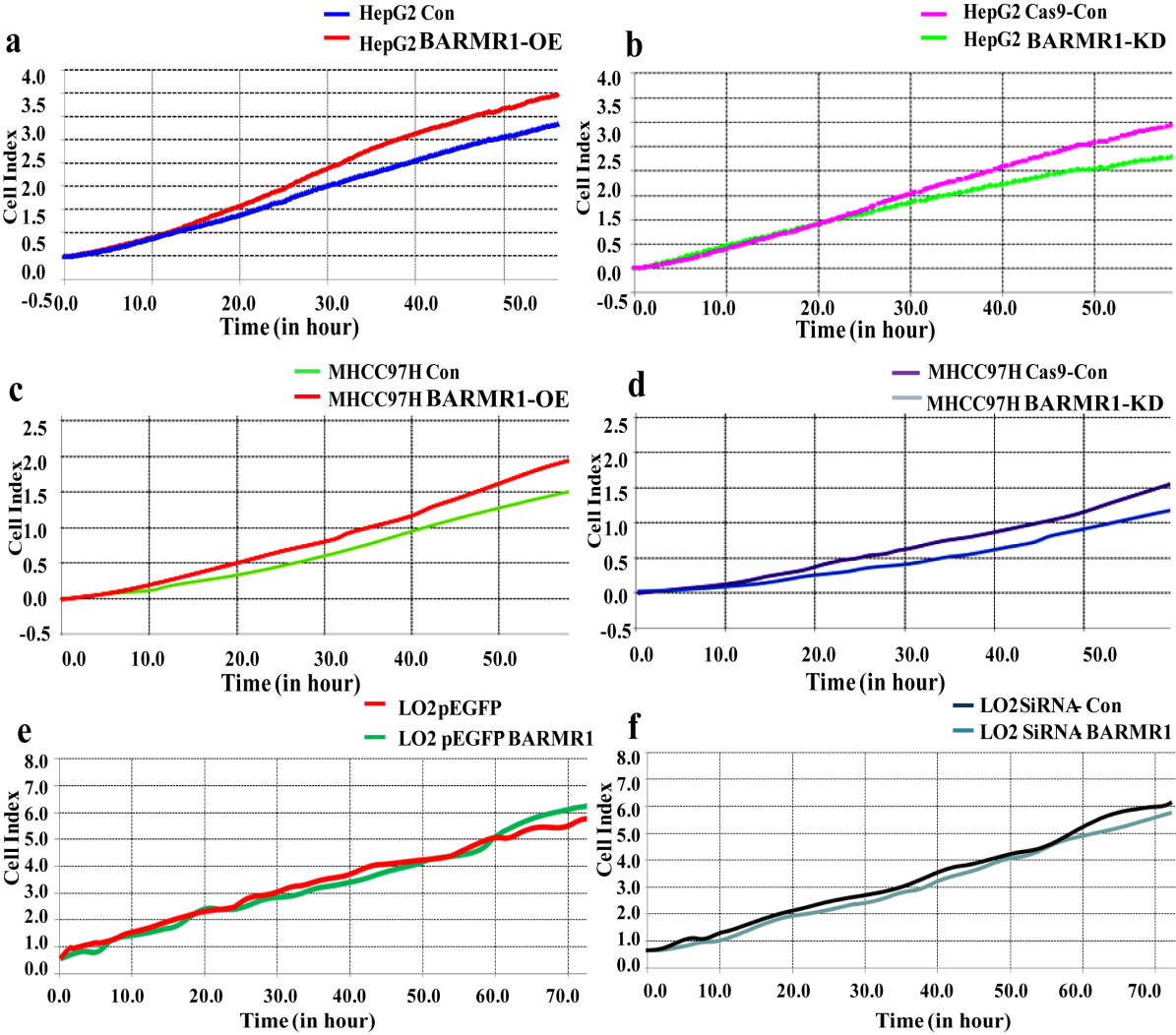


**Fig.S3. BARMR1 is involved in HCC cell proliferation *in vitro***

(a) Real-time monitoring of BARMR1-induced proliferation in HepG2 cells by RTCA (xCELLigence real-time cell analyzer).

(b)Real-time monitoring of BARMR1-induced proliferation in MHCC97H cells by RTCA.

(c)Real-time monitoring of BARMR1-induced proliferation in LO2 cells by RTCA. Each tracing represents the average of three parallel assessments.


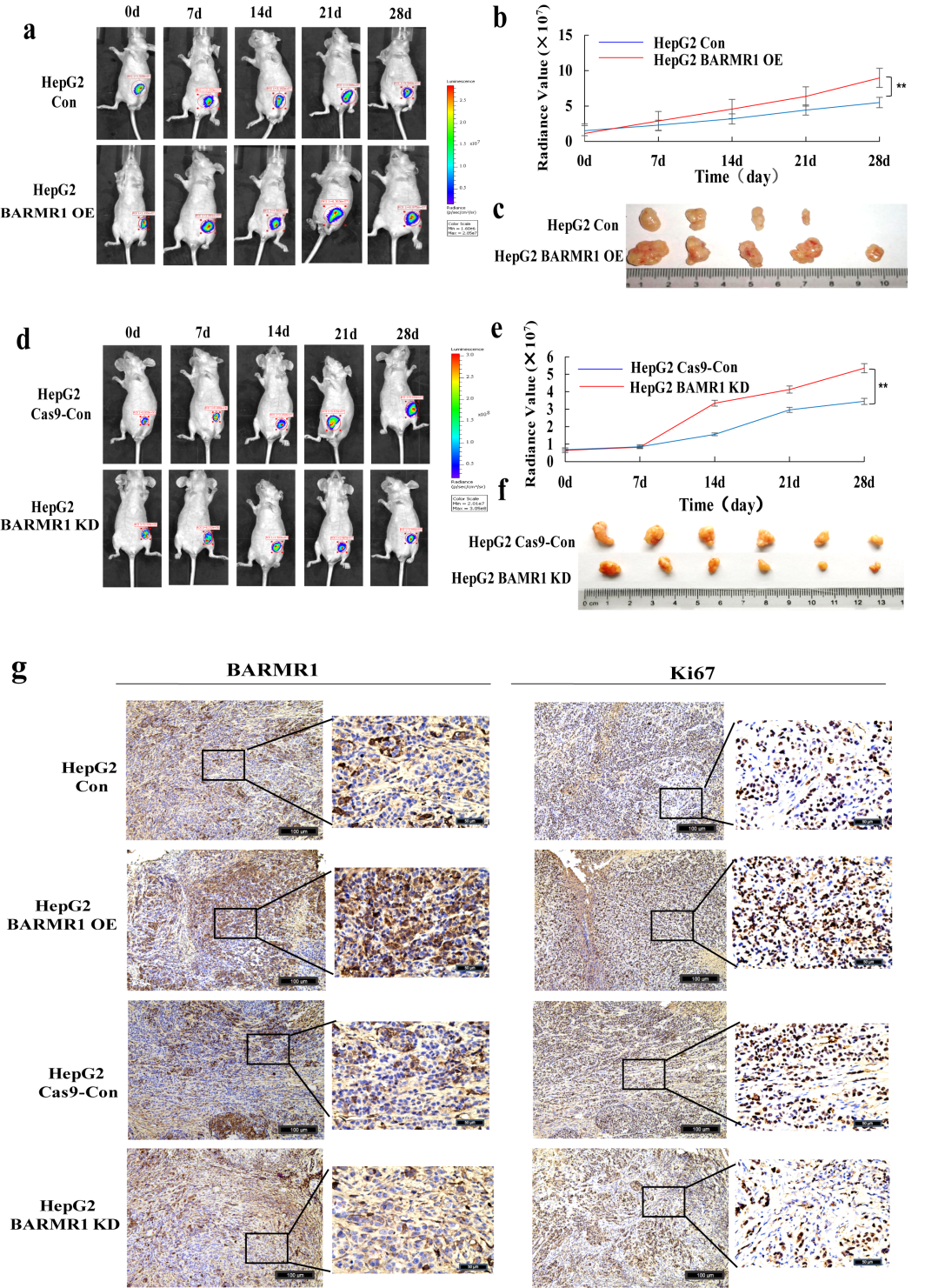


**Fig.S4. BARMR1 is involved cell proliferation in HepG2-Luc cells *in vivo.***

The HepG2-Luc cells with overexpression/knockdown of BARMR1 (1x10^7^ cells/each) were subcutaneously injected into the flanks of nude mice (n ≥ 5). The sizes of neoplasms formed were measured with an in vivo imaging system. The whole animal images were taken every week starting from day 0 until the end of the experiment (day 28).

(a-c) Ectopic expression of BARMR1 accelerated growth of HepG2-Luc cell-derived xenograftsin nude mice as compared to controls. (a) Serial pictures taken at different time points. (b)The changes of fluorescein radiance values of the neoplasms formed from different groups (**p<0.001 vs. control). (c) Representative images of HepG2-Luc cell derived xenograft tumors.

(d-f) Knockdown of BARMR1 decelerated growth of HepG2-Luc cell-derived xenografts in nude mice, serial pictures taken at different time points.(d) Serial pictures taken at different time points. (e)The changes of fluorescein radiance values of the neoplasms formed from different groups (**p<0.001 vs. control). (f) Representative images of HepG2-Luc cell derived xenograft tumors.

(g) Representative images of BARMR1 and Ki67 protein expression in HepG2-Luc cell-derived xenograft tissues in nude mice by immunohistochemistry.


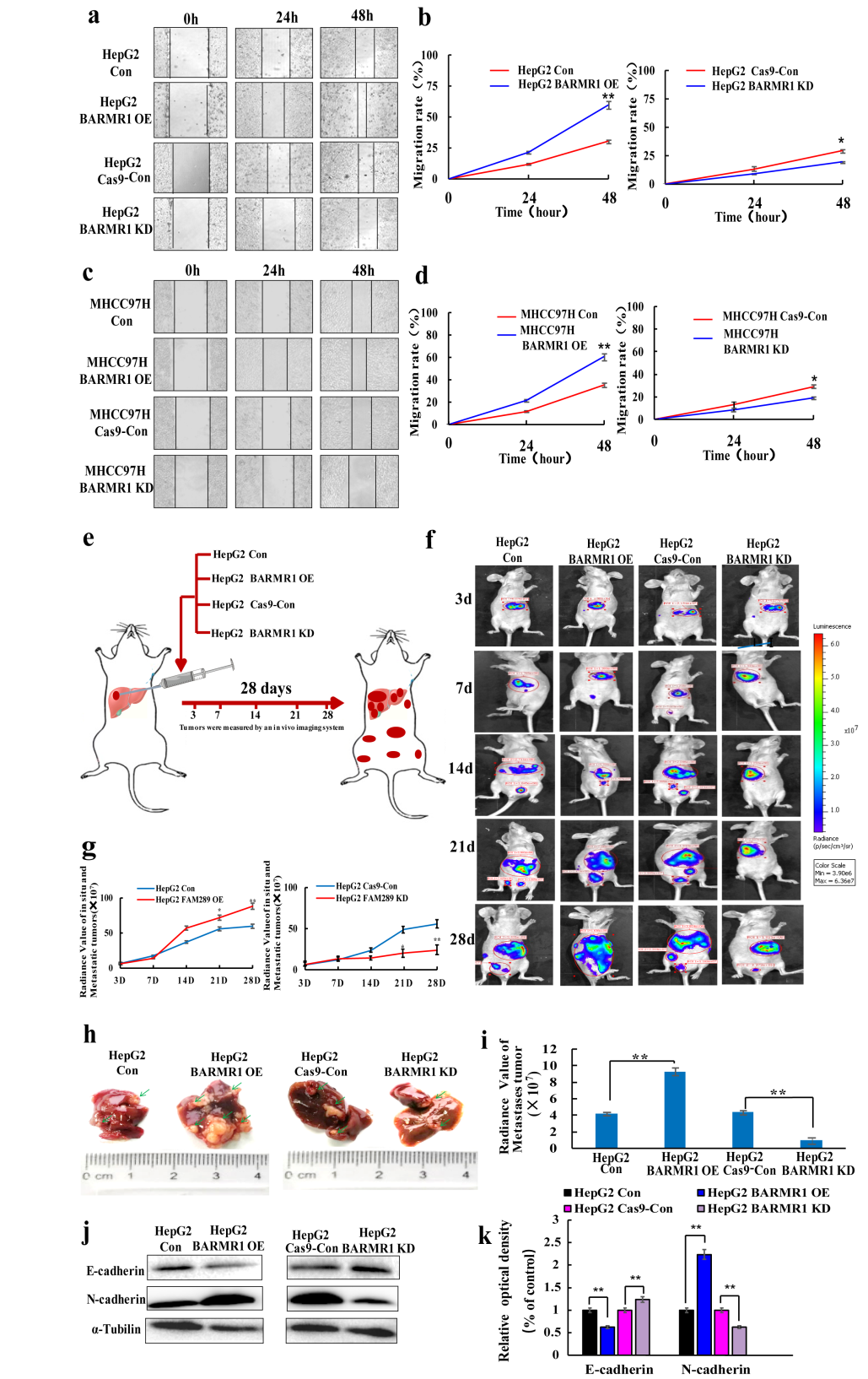


**Fig.S5 BARMR1 is involved in HCC cell migration *in vitro* and *in vivo*.**

(a) The effect of BARMR1 on migrating capacity in HepG2 cells. (a)The representative pictures were taken at 0 h, 24 h and 48 h after scratching. (b)The differences were significant both at 24 h and at 48 h (*p<0.05 and **p<0.001 vs. control).

(b)The effect of BARMR1 on migrating capacity in MHCC97H cells. (c)The representative pictures were taken at 0 h, 24 h and 48 h after scratching. (d)The differences were significant both at 24 h and at 48 h (*p<0.05 and **p<0.001 vs. control).

(c) Schematic model illustrating the administration time and route of HepG2-Luc cells with overexpression/knockdown of BARMR1 in a mouse orthotopic model of metastasis.

(d) The HepG2-Luc cells with overexpression/knockdown of BARMR1 (10 million cells each mouse) were subcutaneously injected into the liver of nude mice (n ≥ 5) and the sizes of orthotopic neoplasms were measured with an in vivo imaging system every week.

(e) The changes of fluorescein radiance values of the neoplasms formed from different groups.

(f) Representative images of HepG2-Luc cell-derived xenograft tumors (h) and relevant metastatic tumors(i) (* p<0.05 and **p<0.001 vs. control).

(g) BARMR1 regulates the expression of metastasis-related proteins. Protein levels of E-cadherin, N-cadherin and CD36 in HepG2 cells with BARMR1 overexpression or knockdown of BARMR1 were detected by western blotting.


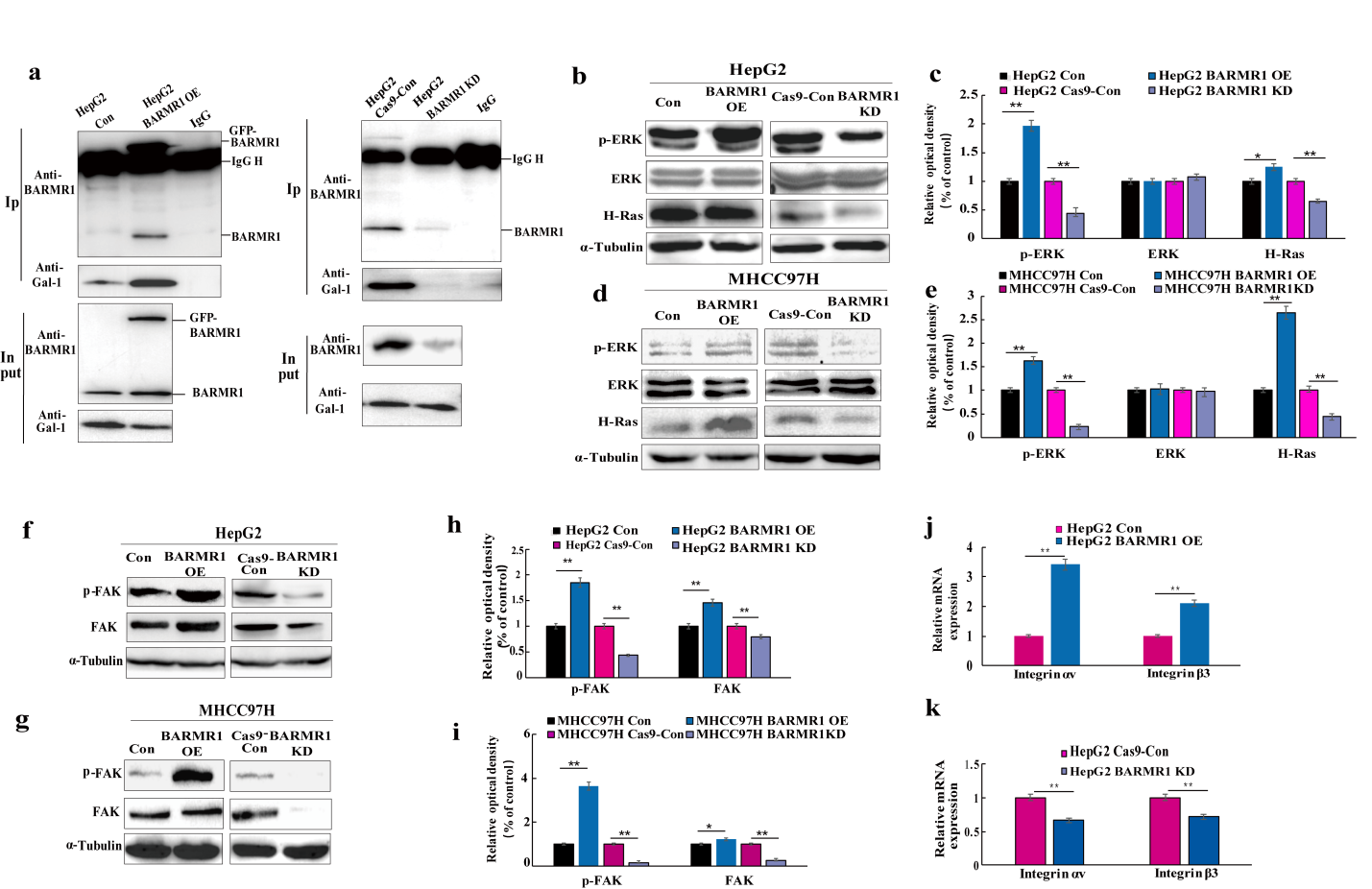


**Fig.S6 BARMR1 activates H-RAS/ERK and αvβ3-integrin/FAK cellular signaling pathways depending on its interaction with Galectin-1**

(a) The interaction of BARMR1 and Gal-1 was examined by immunoprecipitation, co-IP experiment was performed using anti-BARMR1 antibody in HepG2 cells. IgG was used as the negative control. The immunoprecipitates were analyzed via western blotting with anti-BARMR1 and anti-Galectin-1 antibodies.

(b&c) BARMR1 expression level affects the activities of the H-Ras/ERK pathway, protein levels of H-Ras/ERK in HepG2(b) and MHCC97H(c) cells with overexpression or knockdown of BARMR1 were detected by western blotting (**P <0.001 vs. control).

(d&e) BARMR1 regulates the αvβ3-integrin/FAK pathway, the protein levels of FAK and pFAK in HepG2(d) and MHCC97H(e)cells with overexpression or knockdown of BARMR1HepG2 were detected by western blotting (**P <0.001 vs. control).

(f)The expression of αvβ3-integrin was quantified by qRT–PCR in HepG2 cells with overexpression or knockdown of BARMR1.


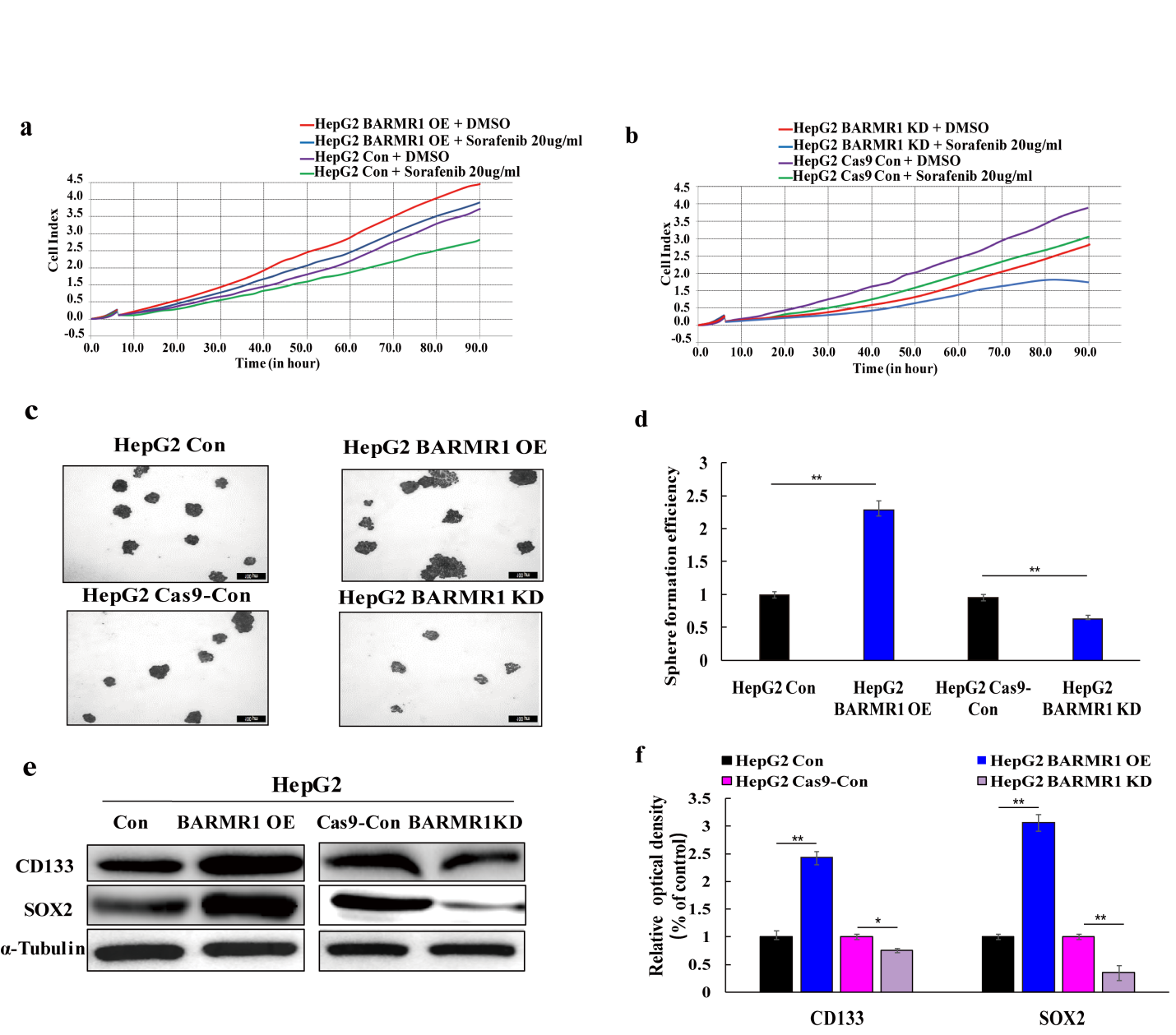


**Fig. S7 BARMR1-mediated sorafenib resistance is through stem-like property acquisition.**

(a) The proliferation of HepG2 cells treaded with sorafenib was analyzed by RTCA. Each tracing represents the average of three parallel assessments.

(b) The in vitro colony formation of HepG2 cells. Images were taken on day 10 under an inverted microscope (**P <0.001).

(c) Cell lysates from HepG2 cells with overexpression or knockdown of BARMR1 were immunoblotted with anti-stem like property factors (SOX2, CD133) (**P <0.001 vs. control).

**Materials and Methods**

**Human HCC samples**

After granted from Ethics Committee of Taihe Hospital (Shiyan, China) and obtained written informed consent, a total of 20 fresh HCC tissues from 20 patients and non-tumorous liver tissues from 6 patients were obtained from Hepatobiliary and Pancreatic Surgery Diagnosis and Treatment Center, Taihe Hospital, Hubei University of Medicine (Shiyan, China). Pathological diagnosis was made according to the histology of tumor specimens or biopsy examined by clinical pathologists. Those tissues were stored in liquid nitrogen until use.

**Pan-Cancer gene copy, expression and patient survival analysis**

Gene copy-number data of publically available patient datasets were obtained from cBioPortal for Cancer Genomics (http://www.cbioportal.org/). TCGA provisional datasets were used for copy-number analysis. BARMR1 expression profiles in patients across cancers were matched with TCGA normal and GTExdata. Overall survival or disease-free survival of cancer patients in TCGA datasets was analyzed using Gene Expression Profiling Interactive Analysis (GEPIA2) (http://gepia2.cancer-pku.cn/).

**Cells and animals**

The human HCC HepG2 and Huh7 were purchased from American Type Culture Collections (ATCC, Manassas, Virginia, USA). MHCC97H, MHCC97L and human liver cell line LO2 were purchased from the Shanghai Cell Bank Type Culture Collection Committee (CBTCCC, Shanghai, China). The human HCC cell lines were maintained as suggested by ATCC. Nude mice (female, 6-8 weeks of age) were purchased from the Model Animal Research Center, Nanjing University (Nanjing, China), and housed in accordance with the National Institutes of Health Guide for the Care and Use of Laboratory Animals of China. The experimental protocols of the present study were approved by the Animal Care Committee of Hubei University of Medicine (Shiyan, China).

**Over-expression of BARMR1 in HepG2 and MHCC97H cells**

HepG2 and MHCC97H cells were cultured till 70-80% confluency and then transfected with CMV-SP6-TALEN-eGFP-289BARMR1 vector using Lipofectamine™3000 transfection reagent (Invitrogen, California，USA) according to manufacturer’s instruction. After 24 hrs of incubation, the transfection medium was replaced with fresh medium containing 2.5μg/ml puromycin to initiate the screening procedure for positive HepG2 and MHCC97H cells. The same culture medium was replaced every third day for 14 days and the cells were used for further experiments.

**Lenti-CRISPRv2/Cas9-mediated down regulation of BARMR1 in HepG2-Luc and MHCC97H cells**

The gRNA-coding cDNAs for targeting BARMR1 gene were designed and synthesized to make the BARMR1-gRNA-Cas9 constructs. The primers including 20 bp target sequence and BsmBI sticky end were annealed and inserted into the lenti-CRISPRv2 plasmid (Genloci Biotechnologies, Jiangsu，China) and digested with BsmBI (NEB, USA). Primer sequences are as follows: sgRNA1, forward, 5'-CACCGACGGGGATTCCAAGGCGCCT-3' and reverse 5'-AAACAGGCGCCTTGGAATCCCCGTC-3'; sgRNA2, forward, 5'-CACCGAAGGCGCCTGGGCGCGCGCT-3' and reverse 5'-AAACAGCGCGCGCCCAG

GCGCCTTC-3'; sgRNA3, forward, 5'-CACCGACGCGTTAATTTCATTCACC-3' and reverse 5'-AAACGGTGAATGAAATTAACGCGTC-3'.

|  |
| --- |

BARMR1 lenti-CRISPRv2 plasmid was used for gene knockdown experiments. To package lentivirus, each lenti-CRISPRv2 plasmid with other components (psPAX2, pMD2G) was transfected into HEK293T cells using Lipofectamine™ 3000 (Invitrogen). The transfected cells were cultured in DMEM containing 5% FBS, 100 U/ml penicillin and 100 μg/ml streptomycin. Culture media were replaced with fresh growth media after 24 hours of initial incubation. 48 hours later, lentiviral particles were concentrated from culture media and filtrated with 0.45 μm filters using Lenti-X Concentrator (Clontech, Mountain View, CA, USA). Aliquots were stored at -80 °C until use. To transduce HepG2-Luc and MHCC97H cells, 1.0×10^5^ cells were plated in each well of a six-well plate, infected with the lentivirus, treated with polybrene for 24 hours and selected by adding 2.5 μg/ml puromycin to the growth medium for 4-6 days. Lentivirus with scrambled sequence was served as the control.

**RNA interference**

The siRNA target sequences used in this study are as follows: BARMR1 siRNA, 5′-CGAUUACCCUGGUUGCACA-3′; GFP siRNA, 5′-CUUACGCUGAGUACUUCGA-3′; All siRNAs were synthesized by RiboBio (Guangzhou, China) and were transfected at a final concentration of 10 nM.

**Reverse transcription PCR**

Total RNA was extracted using TRIzol reagent (Ambion, Austin TX, USA) according to the manufacturer's recommendations and was quantified by UV spectroscopy. To prepare the RNA for PCR analysis, 2 μg total RNA was converted to cDNA using the Fast Quant RT kit with gDNase (Tiangen Biotech, China). Reverse transcription PCR was conducted with a 2×Taq PCR Master mix (Tiangen Biotech, Beijing, China). The PCR reaction was preceded as follows: 95˚C for 30 sec, followed by 30 cycles of 95˚C for 30 sec, 60˚C for 30 sec and 72˚C for 30 sec. The gene expressions were normalized to GAPDH. The primer sequences for the genes were as follows: β-actin, forward, 5'- CCTCGCCTTTGCCGATCC -3' and reverse, 5'- GGATCTTCATGAGGTAGTCA GTC-3'; BARMR1, forward, 5’-GAACGCTCAAACGAAACAA-3’and reverse, 5’- TCACAATGGTCCCATAAGT-3’.

**Western blot analysis**

Cells of each group were collected. The proteins were extracted and separated by SDS-PAGE, and then transferred onto PVDF membranes. After blocking with 1% BSA for 1 hr at room temperature (RT), membranes were incubated with antibodies against GFP, Flag (1:1000, Sigma, St. Louis, Missouri, USA), BARMR1 (FAM92A1 Antibody, Sigma, St. Louis, Missouri, USA), GAPDH，α-Tubulin, ERK, CD133, N-cadherin, E-cadherin, CD36, H-Ras, FAK, Galectin-1, SOX2, (1:1000, Proteintec, Chicago, USA), p-FAK, p-ERK1/2, (Cell Signaling Technology, Boston , USA) at 4˚C overnight. After washing 3 times with TBST, the membranes were incubated with horseradish peroxidase (HRP)-conjugated secondary antibody (1:1000, Beyotime Biotechnology, Shanghai, China) at RT for 1 hr. Then the membranes were washed 3 times with TBST and imaged with a gel imaging system (BIO-RAD, California, USA).

**Co-immunoprecipitation assay**

For the co-immunoprecipitation assay, 293T cells or HepG2 cells at 60-70% confluency in 6-well plates were transfected with a total of 2.5 ug plasmids. Twenty-four hours after transfection, cells were washed once in PBS and lysed in buffer A containing 25mM Tris-HCl, pH7.6, 150mM NaCl, 10% glycerol and 1% Triton X-100, supplemented with a protease inhibitor mixture (Roche Molecular Biochemicals, Stockholm, Sweden). Pre-cleared lysates were subjected to anti-Flag M2 immunoprecipitation (Sigma, St. Louis, Missouri, USA) or protein G magnetic beads (Beyotime Biotechnology, Shang Hai, China) following the manufacturer’s instructions. The beads were washed four times with a lysis buffer and the immunoprecipitates were eluted by 2×SDS sample buffer followed by standard immunoblotting analysis. All the immunoprecipitation assays were performed more than three times and representative results were presented.

**Scratch assay**

The scratch assay was used to observe the cell migration. HepG2-Luc, MHCC97H or LO2 cells were cultured to completely confluent in 6-well plates and a scratch was made across the cell monolayer of each well with a 10μl pipette tip. The cell monolayer was then washed 3 times with D-PBS and incubated in serum free DMEM at 37 °C with 5% CO_2_ for 48 hrs. The widths of the scratch were measured and the percentages of the narrow down scratch were compared at 0 hr, 24 hrs and 48 hrs respectively. The experiment was performed in triplicates.

**Cell viability assay**

The cell viability was also detected by real-time assessment using the xCELLigence real time cell analyzer (RTCA, Roche, USA) as previously described ^16^. HepG2-Luc, MHCC97H or LO2 cells were trypsinized and counted with a hemocytometer using trypan blue exclusion method. Cells were resuspended in culture medium. Background measurements were taken from the wells by adding 100μl of the same medium to the E-Plate 16. A volume of 100 μl of cell suspension (2 x 10^3^ cells) was then added to the wells to make a final volume of 200 μl. All cells were allowed to settle at the bottom of the wells at RT for 15 min followed by an incubation at 37°C in 5% CO_2_ atmosphere. The impedance signals were recorded every 15 min for 72 hrs.

**In vivo models**

Subcutaneous xenograft model: The HepG2-Con-Luc, HepG2-BARMR1-OE-Luc, HepG2-Cas9-Con-Lucand HepG2-BARMR1-KD-Luc cells (1x10^7^cells/each) were subcutaneously injected into nude mice (6 mice/group) separately.

Orthotopic model: The HepG2-Con-Luc, HepG2-BARMR1-OE-Luc, HepG2-Cas9-Con-Luc and HepG2-BARMR1-KD-Luc cells (8x10^6^ cells/each) were injected into nude mouse livers (6mice/group) separately.

The sizes of grafted tumors were measured every 7 days using a small animal image system (IVIS, Caliper Life Sciences, Hopkinton, MA, USA) until the mice died. Each test was conducted 15 min after the anesthetized (with isoflurane) mouse received an intraperitoneal injection of 100μl/20g D-luciferin solution (0.15 mg/ml, Caliper Life Sciences, Hopkinton, MA, USA). The growth rate and size of tumors were analyzed using the radiance value which is proportional to the number of bioluminescence-producing cells.

**Drug treatment**

Nude mice received orthotopic transplantation of HepG2-Luc cells (4x10^6^ cells) on the day 0 and were administered free-form sorafenib daily at a dose of 20 mg/kg body weight (I.P.) until day 14. All control mice received an equal volume of vehicle solution. Tumor development/response to the treatment was monitored by IVIS once per week. The mice were sacrificed 4 weeks after treatment. Tumors and any metastases to liver and retroperitoneum were harvested for further assessments.

**Tumorsphere formation assay**

The HepG2-Luc cells in good growth condition were counted and 1×10^3^ cells were plated into 12-well Ultra Low Cluster plates (Corning, New York, USA). The cells were cultured with a serum-free DMEM/F12 medium containing 100 U/ml penicillin, 100 μg/ml streptomycin, 20 ng/ml epidermal growth factor (EGF) and 10 ng/ml basic fibroblast growth factor (bFGF) (Gibco, Grand Island, NY USA) at 37˚C for 10-14 days. Three replicates were set for each type of cell. The culture medium was replaced according to the cell growth rate and the color changes of the culture medium. The cultures were photographed regularly under a microscope to observe tumor-sphere formation.

**Apoptosis and cell cycle assays**

Cells in early and late apoptotic phase were quantified using an Annexin V-APC/PE double staining assay. Cells were collected and resuspended in 500μL binding buffer at 1 × 10^6^ cells/ml, followed by staining with 5μL Annexin V and 5μL PE in the dark at RT for 15 min. Stained cells were immediately examined using a FACS flow cytometry analyzer (Beckman Coulter, California, USA) with the wavelength emission filters of 488–530 nm for green fluorescence of Annexin V (FL1) and of 488–630 nm for red fluorescence of PI (FL2). For cell cycle assay, 3 × 10^5^ cells/well was seeded into a 6-well plate. After 24 h incubation, the cells were collected and fixed with 75% cold ethanol (1 mL PBS and 3 mL absolute ethanol) at −20 °C for overnight. After that, the cells were incubated with 500μL propidium iodide (PI, 100μg/mL) for 30 min at RT in the dark and analyzed using the FACScan flow cytometer (Becton Dickinson, Franklin Lakes, NJ, USA). The data were analyzed with ModFitLT V2.0 software (Becton Dickinson, Franklin Lakes, NJ, USA). All experiments were performed for three independent times.

**Immunohistochemical staining**

Briefly, 5-μm serial sections were dewaxed in xylene and rehydrated through graded alcohols. Endogenous peroxidases were blocked with 3% H_2_O_2_ for 30 min, and antigens were retrieved by microwaving slides. After cooling and washing, slides were blocked with goat serum for 30 min (1:10; Zymed antibody diluent). The sections were incubated with primary antibodies at 4 °C overnight and incubated with HRP-conjugated secondary antibodies followed by the Liquid DAB Substrate Chromogen System according to the manufacturer’s instruction. The sections were examined under a fluorescence microscope (Olympus, Tokyo Metropolitan, Japan).

**Statistical analysis**

Numeral results were presented as mean ± SD or dot plot. Unpaired two-tailed Student t test were used as appropriated. The Kaplan-Meier analysis was used to estimate overall survival. All statistical analyses were performed with SPSS (version 16.0) and GraphPad (Version 5.0). A p value less than 0.05 was considered to be a statistical significance.
