# Supplementary figures and images for: BARMR1-mediated sorafenib resistance is derived through stem-like property acquisition by activating integrin-FAK signaling pathways
Source: Signal Transduct Target Ther. 2020 Jun 12;5:97. doi: 10.1038/s41392-020-0189-8 (PMC7293271; doi:10.1038/s41392-020-0189-8)

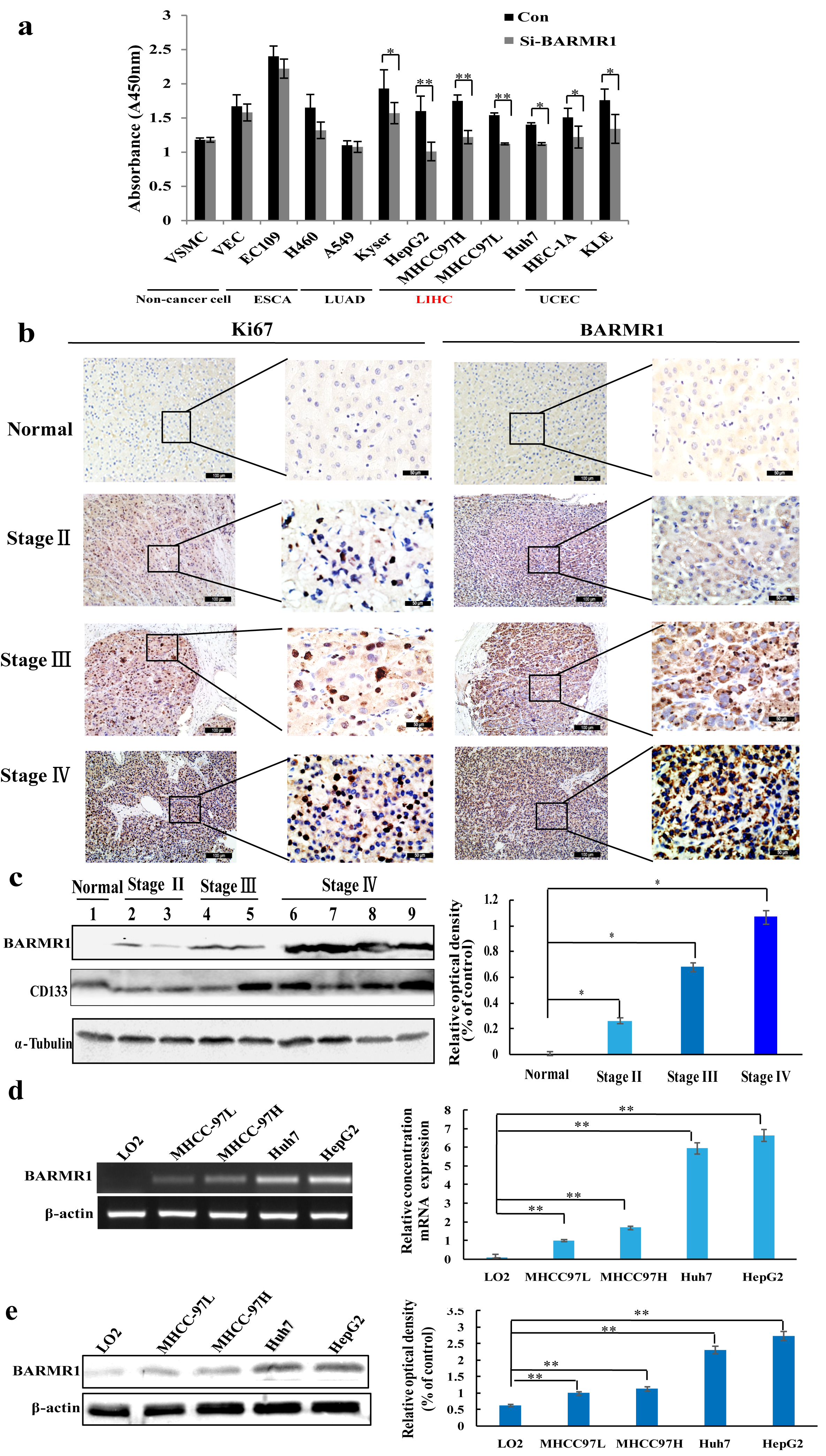

Supplement: Supplementary file 3 — SupplementalFigure s2 [file 41392_2020_189_MOESM3_ESM.tif]

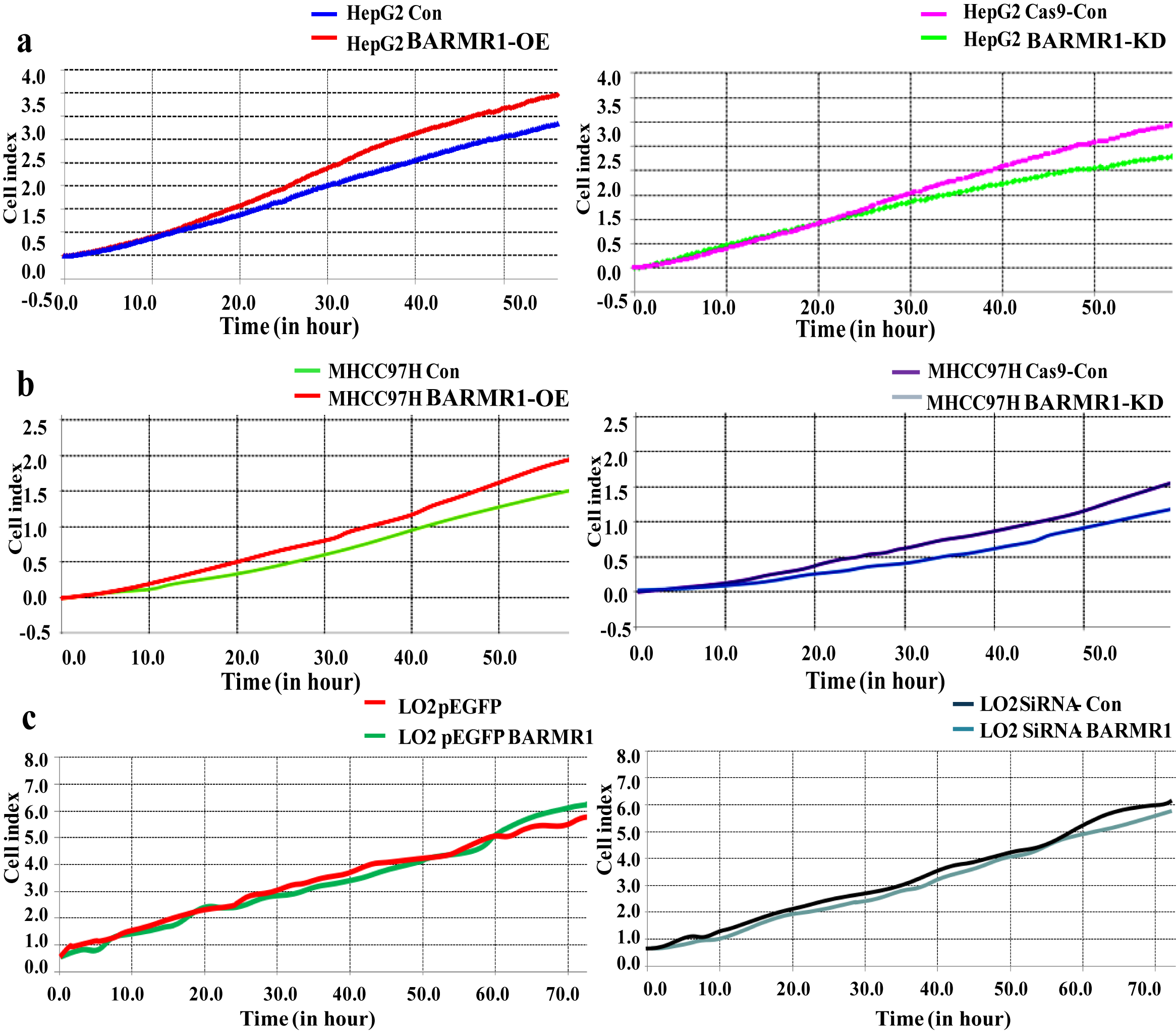

Supplement: Supplementary file 4 — SupplementalFigure s3 [file 41392_2020_189_MOESM4_ESM.tif]

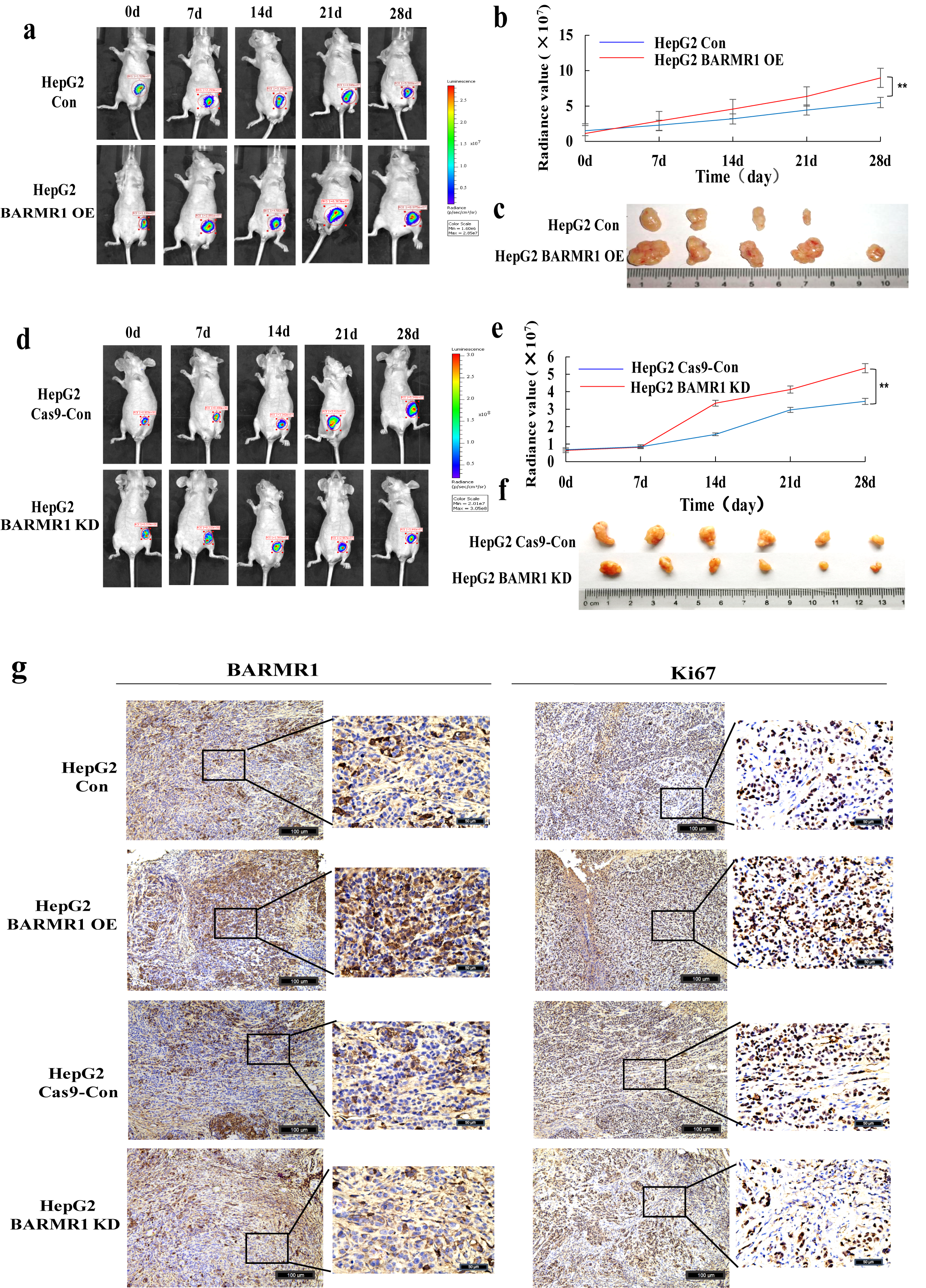

Supplement: Supplementary file 5 — SupplementalFigure s4 [file 41392_2020_189_MOESM5_ESM.tif]

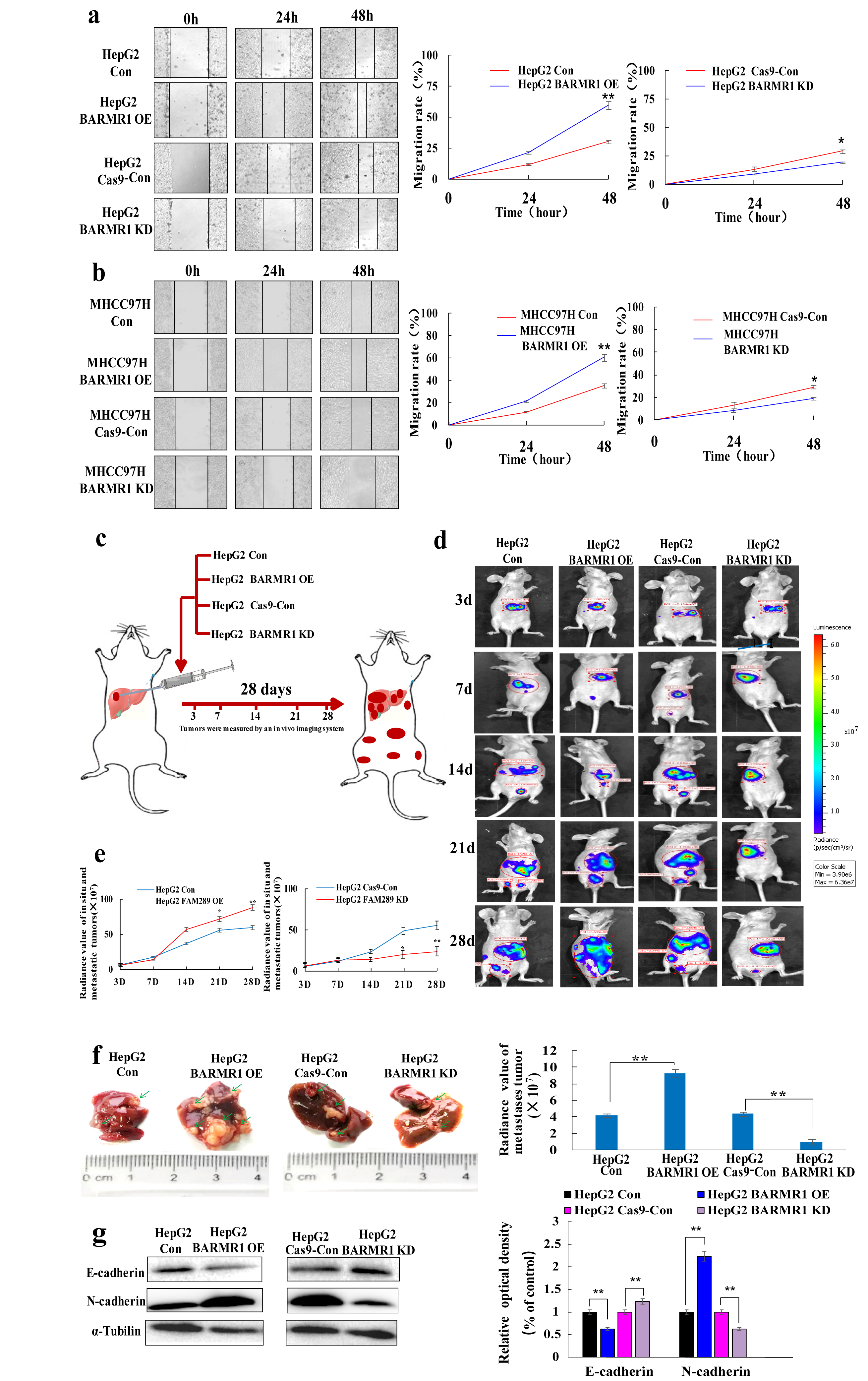

Supplement: Supplementary file 6 — SupplementalFigure s5 [file 41392_2020_189_MOESM6_ESM.tif]

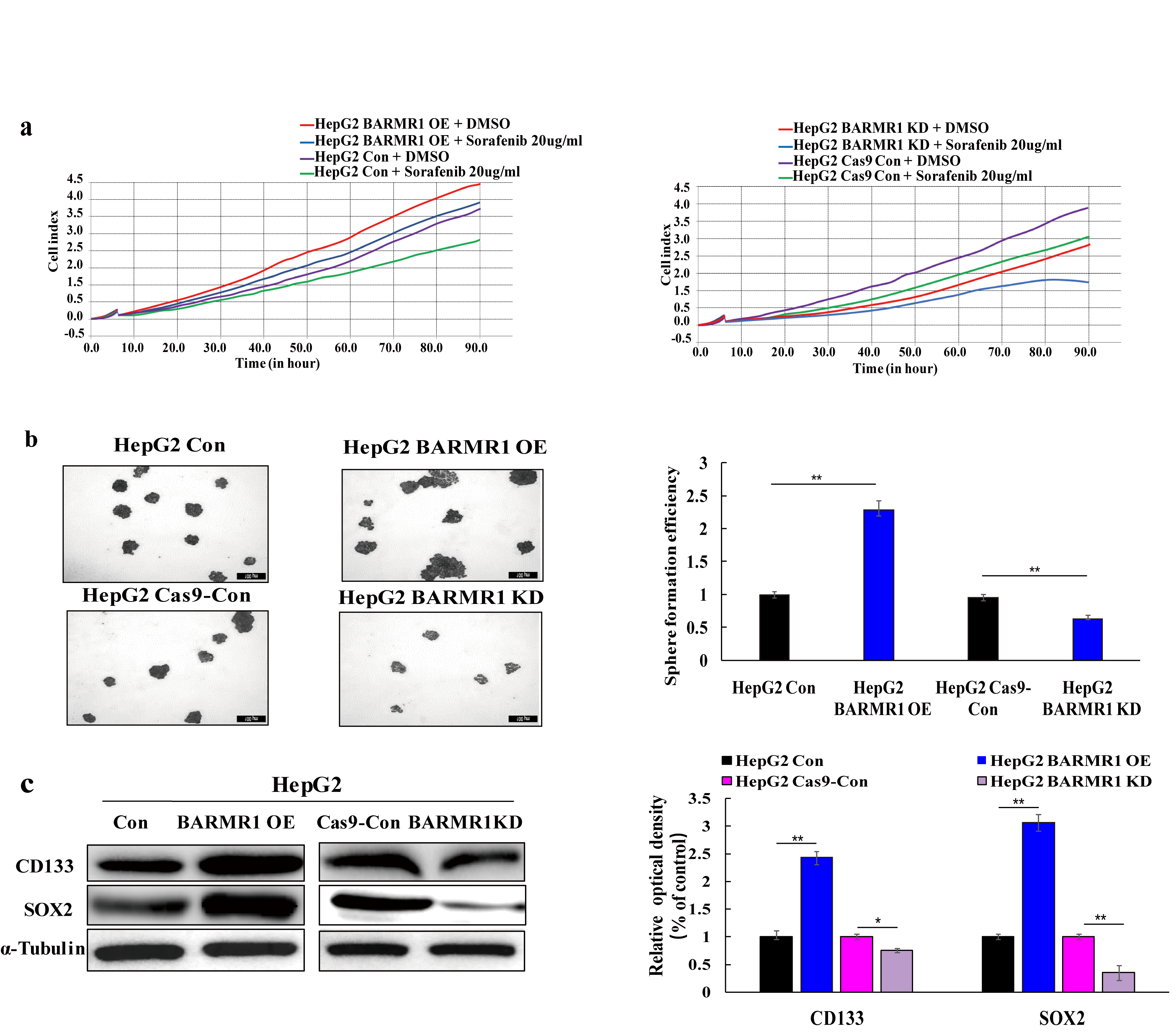

Supplement: Supplementary file 8 — SupplementalFigure s7 [file 41392_2020_189_MOESM8_ESM.tif]
